# Baker’s Yeast Sensitizes Metastatic Breast Cancer Cells to Paclitaxel In Vitro

**DOI:** 10.1177/1534735417740630

**Published:** 2017-11-21

**Authors:** Nariman K. Badr El-Din, Ashraf Z. Mahmoud, Tahia Ali Hassan, Mamdooh Ghoneum

**Affiliations:** 1 University of Mansoura, Mansoura, Egypt; 2 Drew University of Medicine and Science, Los Angeles, CA, USA

**Keywords:** *Saccharomyces cerevisiae*, apoptosis, paclitaxel, breast cancer, 4T1

## Abstract

Our earlier studies have demonstrated that phagocytosis of baker’s yeast (*Saccharomyces cerevisiae*) induces apoptosis in different cancer cell lines in vitro and in vivo. This study aimed to examine how baker’s yeast sensitizes murine and human breast cancer cells (BCC) to paclitaxel in vitro. This sensitizing effect makes lower concentrations of chemotherapy more effective at killing cancer cells, thereby enhancing the capacity of treatment. Three BCC lines were used: the metastatic murine 4T1 line, the murine Ehrlich ascites carcinoma (EAC) line, and the human breast cancer MCF-7 line. Cells were cultured with different concentrations of paclitaxel in the presence or absence of baker’s yeast. Cell survival and the IC_50_ values were determined by MTT assay and trypan blue exclusion method. Percent of DNA damage, apoptosis, and cell proliferation were examined by flow cytometry. Yeast alone and paclitaxel alone significantly decreased 4T1 cell viability postculture (24 and 48 hours), caused DNA damage, increased apoptosis, and suppressed cell proliferation. Baker’s yeast in the presence of paclitaxel increased the sensitivity of 4T1 cells to chemotherapy and caused effects that were greater than either treatment alone. The chemosensitizing effect of yeast was also observed with murine EAC cells and human MCF-7 cells, but to a lesser extent. These data suggest that dietary baker’s yeast is an effective chemosensitizer and can enhance the apoptotic capacity of paclitaxel against breast cancer cells in vitro. Baker’s yeast may represent a novel adjuvant for chemotherapy treatment.

## Introduction

Chemotherapy is currently the mainstay of treatment for most types of cancer, with agents exerting their anticancer effect by inducing apoptosis.^1,2^ One such drug currently in use is paclitaxel. Paclitaxel is most often used for the treatment of breast cancer, ovarian cancer, non–small cell lung cancer, and AIDS-related Kaposi’s sarcoma.^3^ Paclitaxel is able to induce the mitochondrial apoptotic pathway in various cancer cell types. Paclitaxel induces the mitochondrial permeability transition, triggering the release of prodeath molecules (Bax and Bad, which then inactivate Bcl-2 or Bcl-x_L_) and activating caspases, which then induce apoptosis of neoplastic cells.^4-6^ Chemotherapy drugs can be effective in treating cancer; however, many chemotherapeutic agents exhibit dose-limiting toxicities, which can cause congestive heart failure, myelosuppression, neurotoxicity, immune-suppression, and mutagenic and carcinogenic effects.^7-10^ Many attempts have been made to circumvent the toxic effects of chemotherapy, including using chemosensitizers, which make tumor cells more sensitive to the effects of chemotherapy and therefore require lower doses of the toxic chemotherapeutic drugs. In the past 40 years, research has focused on the development of potent therapies to circumvent multidrug resistance (MDR), and several chemosensitizing agents have been discovered which enhance the cytotoxic effect of chemotherapy drugs in cancer cells; these include the calcium blockers diltiazem, the biscoclaurine alkaloid cepharanthine, and verapamil^11-14^; the anti-arrhythmic agent quinidine^15^; and the synthetic isothiocyanate derivative E-4IB.^16^ However, these agents are also toxic. Calcium antagonist poisoning is well documented, and other side effects may include dizziness, headache, redness in the face, fluid buildup in the legs and ankles, abnormal heart rate, constipation, and gingival overgrowth.^17,18^

Recently, several researchers have focused on screening for nontoxic natural modulators to overcome ABC transporter-mediated MDR. Two natural products, curcumin and flavonoids, have been extensively studied in the context of modulation of MDR transporter expression.^19,20^ In addition, work from our laboratory has introduced a novel, natural, dietary product, arabinoxylan rice bran (MGN-3/Biobran), which when combined with chemotherapy allowed for lowering the drug concentration used during treatment, thereby reducing the toxicity of chemotherapy while maintaining potency against cancer cells in vitro and in vivo.^21-23^ In this study, we evaluated the ability of another dietary product, baker’s and brewer’s yeast, *Saccharomyces cerevisiae*, to sensitize cancer cells to chemotherapy in vitro. *S. cerevisiae* is a potent apoptotic agent against cancer cells. Cancer cells undergo apoptosis upon phagocytosis of *S. cerevisiae*. Baker’s yeast can induce apoptosis in several human cancer cell lines, including breast, tongue, and colon cells in vitro.^24-27^ Yeast can also exert anticancer effects in nude mice bearing human breast cancer^28,29^ and in Swiss albino mice bearing Ehrlich carcinoma.^30^

Our results reveal that *S. cerevisiae* induces anticancer effects in murine and human breast cancer cells and, when combined with paclitaxel, induces killing of a greater number of cancer cells than either yeast or paclitaxel used alone. These results suggest that baker’s yeast may be used as an adjuvant during chemotherapy treatment and may have clinical implications for the treatment of breast cancer.

## Materials and Methods

### Drugs and Chemicals

Paclitaxel was purchased from Bristol-Myers Squibb Inc (Princeton, NJ, USA). It was supplied with initial concentration of 100 mg/16.7 mL. Each milliliter of sterile nonpyrogenic solution contains 6 mg paclitaxel, 527 mg of purified Cremophor EL (polyoxyethylated castor oil), and 49.7% (v/v) dehydrated alcohol, USP. RPMI-1640 supplemented with 10% fetal calf serum (FCS), 3-(4,5-dimethylthiazol-2-yl)-2,5-diphenyltetrazoliumbromide (MTT) from Sigma-Aldrich.

### Preparation of Saccharomyces cerevisiae

Commercially available baker’s and brewer’s yeast, *S. cerevisiae*, was used in suspensions that were washed once with phosphate-buffered saline (PBS). It was then incubated for 1 hour at 90°C to kill the yeast and washed 3 times with PBS. Quantification was carried out using a hemocytometer, and cell suspensions were adjusted to 1 × 10^4^, 1 × 10^5^, 1 × 10^6^, 1 × 10^7^, 1 × 10^8^, and 1 × 10^9^ cells/mL.

### Breast Cancer Cell Lines and Culture Conditions

Three breast cancer cell (BCC) lines were used in the study: the highly metastatic murine 4T1 line; the murine Ehrlich ascites carcinoma (EAC) cell line, a mammary adenocarcinoma; and the human nonmetastatic breast cancer MCF-7 line. 4T1 and MCF-7 cells were purchased from the American Tissue and Culture Collection (ATCC; Manassas, VA, USA). 4T1 and MCF-7 cells were maintained in our laboratory in a complete medium that consisted of RPMI-1640, supplemented with 10% FCS, 2 mM glutamine, and 100 µg/mL streptomycin and penicillin.

We chose MCF-7 as our human BCC line since MCF-7 cells have been found to be more susceptible to yeast-induced apoptosis as compared with other human BCC lines, including ZR-75 cells and HCC70 cells.^24^ Furthermore, MCF-7 cells proved to be potent phagocytic cells as exemplified by their ability to rapidly engulf, digest, and fragment yeast cells with lysosomal encirclement of the engulfed yeast cells.^25,29^

### Preparation of Ehrlich Ascites Carcinoma Cells

The transplantable murine tumor cell line, namely EAC cells, was obtained from the National Cancer Institute, Cairo University, Egypt. The EAC cells were maintained in the ascitic form in vivo in Swiss albino mice by means of sequential intraperitoneal transplantation of 2 × 10^6^ cells/mouse after every 10 days. Ascitic fluid was drawn out from EAC-bearing mouse 8 days after transplantation from the peritoneal cavity by aspirating the ascitic fluid into a sterile isotonic saline solution. The freshly drawn fluid was diluted with ice-cold sterile PBS (0.2 M, pH 7.4), and the tumor cell count was adjusted to 2 × 10^6^ cells/mL by sterile PBS immediately before the studies.

### Effect of Paclitaxel Plus Yeast on Growth of Breast Cancer Cells

#### Drug Sensitivity Assay

Drug sensitivity was determined by using a colorimetric MTT assay. Cancer cells (1 × 10^4^ cells/well) were seeded in 96-well plates and cultured in triplicate with different concentrations of yeast (1 × 10^4^ to 1 × 10^9^ cells/mL) and in the presence or absence of paclitaxel at different concentrations (1 × 10^−6^ to 1 × 10^−1^ M/L). The final volume of medium in each well after the addition of yeast or paclitaxel was 200 µL. The cultures were incubated at 37°C for 24 and 48 hours, after which 50 µg of MTT were added to each well, and the cultures were incubated for an additional 4 hours. The plates were then centrifuged, the medium was carefully removed, the formazan crystals solubilized with acid alcohol, and the plates were read at 590 nm using an ELISA plate reader (Molecular Devices, Menlo Park, CA, USA). The 50% inhibitory concentration (IC_50_) was determined as the drug concentration resulting in a 50% reduction of cell viability. The IC_50_ was determined by plotting the logarithm of the drug concentration versus the survival rate of the treated cells.

#### Trypan Blue Exclusion Method

In sterile test tubes, the cells and chemicals were added with the aforementioned concentrations of yeast, paclitaxel, and both yeast plus paclitaxel in triplicates. Cells were incubated for 24 and 48 hours at 37°C in a humidified atmosphere of 5% CO_2_ in sterile medium. Viable cells were counted by trypan blue exclusion using hemocytometer. Then the percentage of live cells was obtained by dividing the viable cells by the total number of cells. All experiments were repeated in triplicates.

### Flow Cytometric Analysis for Apoptosis, DNA Damage, and Cell Proliferation

Quantitative detection of apoptosis, DNA damage, and cell proliferation in 4T1 cells treated with yeast with and without paclitaxel was simultaneously determined by multicolor flow cytometric analysis using the Apoptosis, DNA Damage and Cell Proliferation Kit specific for incorporated bromodeoxyuridine (BrdU), phosphorylated H2AX (#H2AX), and cleaved poly ADP ribose polymerase (PARP) (BD Biosciences Pharmingen, San Diego CA, USA). Following the manufacturer’s instructions, cells were cultured in a CM or with different concentrations of yeast (1 × 10^7^ cells/mL) and (1 × 10^9^ cells/mL) with and without paclitaxel (1 × 10^3^ M) for 24 or 48 hours. Ten microliters of BrdU working solution (1 mM BrdU in 1× [DPBS]) was added to each milliliter of tissue culture medium (the cell culture density was approximately 1 × 10^6^ cells/mL); following this, the cells were incubated for 30 minutes on ice. Cells were washed by adding 1 mL of staining buffer/tube and centrifuged (5 minutes) at 250 × *g*, and the supernatant was discarded. Cells were fixed with 100 µL of BD Cytofix/Cytoperm Fixation/Permeabilization Solution per tube and incubated for 30 minutes at room temperature. Afterward, cells were washed with 1 mL of 1× BD Perm/Wash Buffer, centrifuged, and the supernatant was discarded. Cells were incubated in 100 µL of BD Cytofix/Cytoperm Plus Permeabilization Buffer/ tube for 10 minutes in ice, washed, and then refixed for 5 minutes. One hundred microliters of diluted DNase were added to cells, which were incubated for 1 hour at 37°C and then washed. Cells were resuspended with 20 µL wash buffer plus PerCP-Cy 5.5 mouse anti-BrdU (5 µL/test), Alexa Fluor 647 mouse anti-H2AX (pS139) (5 µL/test), PE anti-cleaved PARP (Asp214) (5 µL/test) for 20 minutes in the dark and then washed. Cells were resuspended in staining buffer for analysis by fluorescence-activated cell sorting (FACSCalibur; BD Biosciences, San Jose, CA, USA) using CellQuest 3.3 software.^31,32^

### Statistical Analysis

Values are reported as the mean ± standard error (mean ± SE), and data were analyzed using 1-way analysis of variance followed by post hoc tests for multiple comparisons. A *P* value less than .05 was considered statistically significant.

## Results

### Cytotoxicity of Yeast and Paclitaxel on Breast Cancer Cell Lines

Cytotoxicity of yeast plus paclitaxel was examined against three BCC lines: the highly metastatic murine 4T1 line, the murine EAC cell line, and the human MCF-7 cell line. BCCs were cultured with paclitaxel at different concentrations (10^−6^-10^−1^ M/L) in the presence or absence of yeast at different concentrations (10^4^-10^9^ cells/mL). Results were evaluated with 2 different methods (MTT assay and Trypan blue exclusion method) at 24 and 48 hours incubation time before cell survival and the IC_50_ values were determined.

#### 4T1 Cells

**Figure 1.**
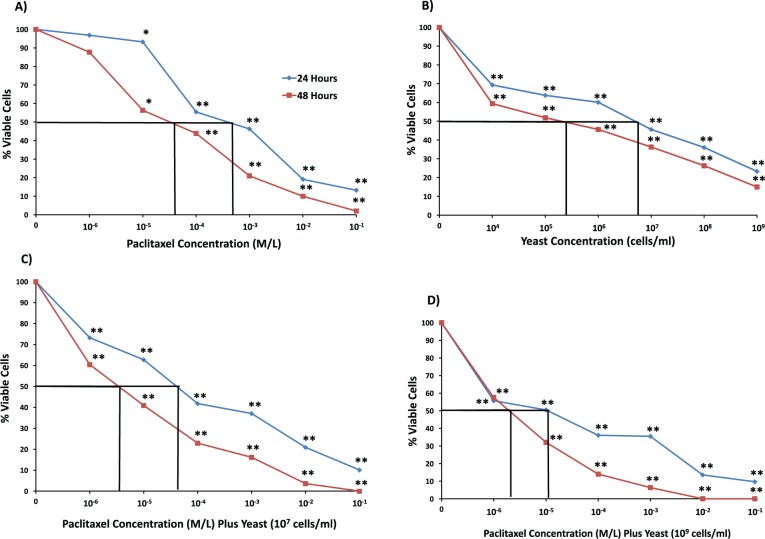
Effect of paclitaxel and yeast on the growth and viability of 4T1 cells as assessed by MTT assay. 4T1 cells were exposed for 24 and 48 hours to the following treatments: (A) paclitaxel alone, (B) yeast alone (1 × 10^4^ to 1 × 10^9^ cells/mL), (C) paclitaxel plus yeast (1 × 10^7^ cells/mL), and (D) paclitaxel plus yeast (1 × 10^9^ cells/mL). Data are the mean ± SE of 2 experiments performed in triplicate. *P < .05, **P < .01 and was considered as statistically significant.

4T1 cells were incubated for 48 hours with paclitaxel and/or yeast, and cell survival was examined by MTT assay and IC_50_ values were also determined (Figure 1A-D). Paclitaxel treatment alone (10^−6^-10^−1^ M/L) caused a decrease in 4T1 cell survival with IC_50_ (5 × 10^−5^ M/L) (Figure 1A). Data depicted in Figure 1B show that yeast treatment alone (10^4^-10^9^ cells/mL) resulted in decreasing the cell survival with IC_50_ (2 × 10^5^ cells/mL). On the other hand, data in Figure 1C show that the cytotoxicity of yeast at low concentration of 10^7^ cells/mL in combination with paclitaxel at different concentrations (10^−6^-10^−1^ M/L) resulted in a significant decrease of 4T1 cell survival with IC_50_ (5 × 10^−6^ M/L). The cytotoxic effect of yeast at higher concentration of 10^9^ cells/mL in combination with paclitaxel became more remarkable with IC_50_ (2 × 10^−6^ M/L) (Figure 1D). Similar results were obtained to a lesser extent at 24 hours. Similar results were noticed when Trypan blue exclusion method was used to determine the levels of toxicity by yeast and paclitaxel against 4T1 cells (data not shown).

#### EAC Cell Line

**Figure 2. fig2-1534735417740630:**
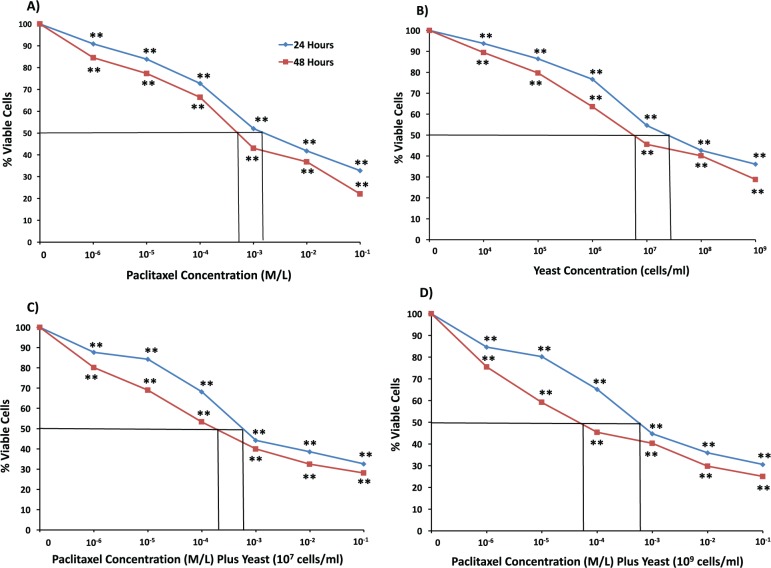
Effect of paclitaxel and yeast on the growth and viability of Ehrlich ascites carcinoma (EAC) cells as assessed by MTT assay. EAC cells were exposed for 24 and 48 hours to the following treatments: (A) paclitaxel alone, (B) yeast alone (1 × 10^4^ to 1 × 10^9^ cells/mL), (C) paclitaxel plus yeast (1 × 10^7^ cells/mL), and (D) paclitaxel plus yeast (1 × 10^9^ cells/mL). Data are the mean ± SE of 2 experiments performed in triplicate. **P < .01 and was considered as statistically significant.

Data in Figure 2A-D show that the combination of yeast with paclitaxel induces higher cytotoxic effects on EAC cells than paclitaxel alone. The decrease in EAC cell survival postexposure to different treatments for 48 hours showed IC_50_ = 6.86 × 10^−4^ M/L for paclitaxel alone (Figure 2A), and IC_50_ = (7 × 10^6^ cells/mL) for yeast alone (Figure 2B). When paclitaxel was combined with yeast (10^7^ cells/mL), IC_50_ decreased to 3 × 10^−4^ M/L) (Figur 2C) and to 6 × 10^−5^ M/L) for 10^9^ cells/mL of yeast (Figure 2D). Similar results, to a lesser extent, were obtained with yeast alone at 24 hours. Also, similar results were noticed when the Trypan blue exclusion method was used (data not shown).

#### MCF-7 Cell Line

**Figure 3.**
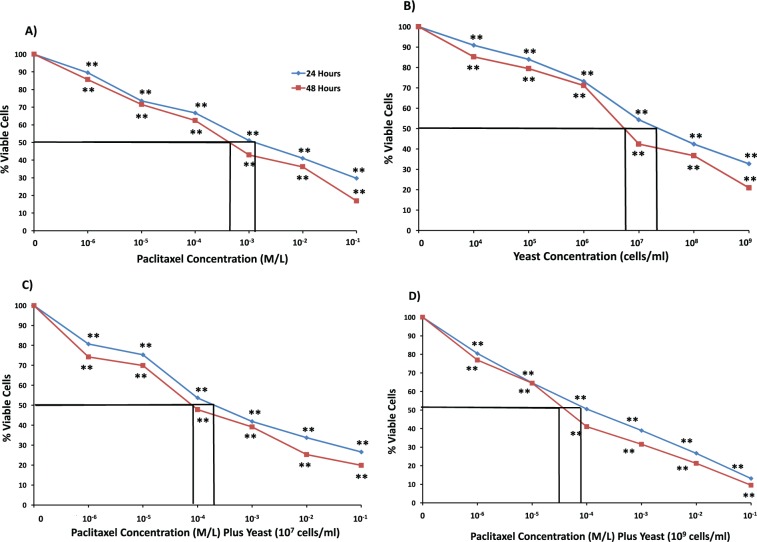
Effect of paclitaxel and yeast on the growth and viability of MCF-7 cells as assessed by MTT assay. MCF-7 cells were exposed for 24 and 48 hours to the following treatments: (A) paclitaxel alone, (B) yeast alone (1 × 10^4^ to 1 × 10^9^ cells/mL), (C) paclitaxel plus yeast (1 × 10^7^ cells/mL), and (D) paclitaxel plus yeast (1 × 10^9^ cells/mL). Data are the mean ± SE of 2 experiments performed in triplicate. **P < .01 and was considered as statistically significant.

The combined effect of paclitaxel and yeast also yielded a higher cytotoxic effect against human breast MCF-7 cells than either treatment alone. Results in Figure 3A and B show that the decrease in MCF-7 cell survival postexposure to different treatments for 48 hours was IC_50_ = 6 × 10^−4^ M/L for paclitaxel alone, and IC_50_ = 6.86 × 10^6^ cells/mL for yeast alone, respectively. When the 2 agents were combined, a significant decrease of MCF-7 cell survival was noticed with IC_50_ = 9 × 10^−5^ M/L for 10^7^ cells/mL yeast (Figure 3C), and IC_50_ = 4 × 10^−5^ M/L for 10^9^ cells/mL yeast (Figure 3D). Similar results were obtained to a lesser extent at 24 hours. Also, similar results were noticed when Trypan blue exclusion method was used to determine the levels of toxicity by yeast and paclitaxel against MCF-7 cells (data not shown).

### Flow Cytometry Analysis for the Evaluation of Cell Proliferation, DNA Damage, and Apoptosis

Data in Figures 1 [Fig fig2-1534735417740630]-3 showed different responses among cell lines toward the cytotoxic effect of paclitaxel, yeast, and yeast plus paclitaxel. We observed the following pattern of sensitivity toward the cytotoxic effect of different treatments: 4T1 > MCF-7 > EAC cells, with 4T1 cells proving to be the most responsive. Therefore, the highly metastatic 4T1 cells were used to examine more detailed effects of different treatments, including DNA damage, apoptosis, and cell proliferation.

#### DNA Damage of 4T1 Cells

**Figure 4.**
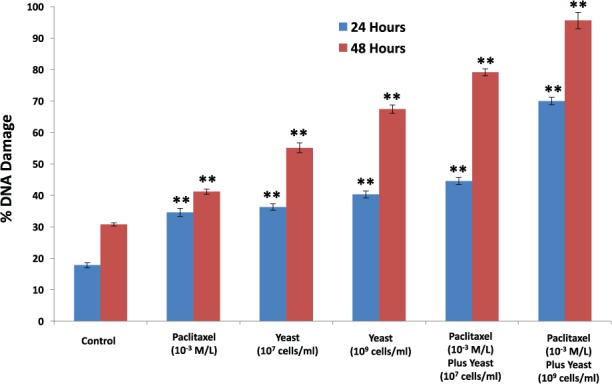
DNA damage to 4T1 cells. The effect of paclitaxel (1 × 10^−3^ M/L), and 2 different concentrations of yeast (1 × 10^7^ and 1 × 10^9^ cells/mL), and paclitaxel plus yeast on DNA damage to 4T1 cells was examined. DNA damage in 4T1 cells was assessed using flow cytometry. Data represent the mean ± SE of 2 experiments performed in triplicate. **P < .01 and was considered as statistically significant.

The effect of yeast at 2 different concentrations (1 × 10^7^ and 1 × 10^9^ cells/mL) and/or paclitaxel (1 × 10^−3^ M/L) on percentage of DNA damage of 4T1 cells was examined. Data in Figure 4 show that treatment of 4T1 cells with paclitaxel alone significantly increased the percentage of DNA damage (94.3%, *P <* .01), as compared with control untreated 4T1 cells. Treatment with yeast at concentrations (1 × 10^7^ and 1 × 10^9^ cells/mL) resulted in 104.1% and 126.5% (*P* < .01), relative to control untreated cells, respectively. On the other hand, exposure of 4T1 cells to both paclitaxel plus yeast resulted in a marked increase in the percentage of DNA damage that was higher than with either agent alone. The chemosensitizing effect of yeast was significant at 24 hours and further increased at 48 hours.

#### Apoptosis of 4T1 Cells

**Figure 5.**
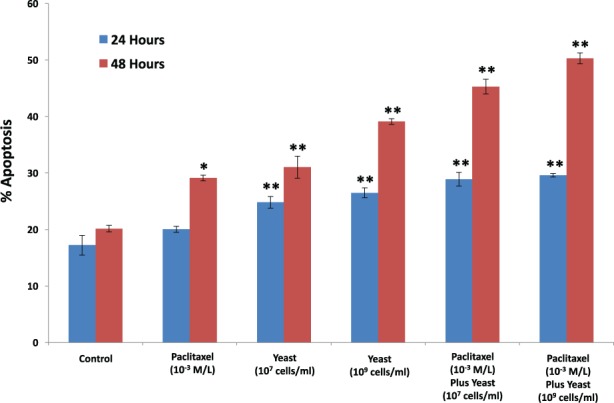
Apoptosis of 4T1 cells. The effect of paclitaxel alone (1 × 10^−3^ M/L), and 2 different concentrations of yeast (1 × 10^7^ and 1 × 10^9^ cells/mL), and paclitaxel plus yeast on apoptosis of 4T1 cells at 24 and 48 hours was examined. The percentage of apoptosis of 4T1 cells was assessed using flow cytometry. Data represent the mean ± SE of 2 experiments performed in triplicate. *P < .05, **P < .01 and was considered as statistically significant.

The effect of yeast at 2 different concentrations (1 × 10^7^ and 1 × 10^9^ cells/mL) and/or paclitaxel (1 × 10^−3^ M/L) on the percentage of 4T1 cell apoptosis was examined. Data in Figure 5 show that treatment with paclitaxel alone increased 4T1 cell apoptosis at 48 hours (44.34%, *P* < .05). Yeast, in a dose-dependent manner, significantly enhanced apoptosis of 4T1 cells (53.87%, *P* < .01) and (93.95%, *P* < .01) at concentrations 1 × 10^7^ and 1 × 10^9^ cells/mL, respectively. However, exposure of 4T1 cells to yeast plus paclitaxel resulted in a higher percentage of 4T1 cell apoptosis: 124.7% (*P* < .01) at yeast concentration 1 × 10^7^ cells/mL and 149.5% (*P* < .01 level) at yeast concentration 1 × 10^9^ cells/mL as compared to control. The apoptotic effect of combined agents was higher than that with either agent alone. The apoptotic effect of yeast was significant at 24 hours and further increased at 48 hours.

#### Proliferation of 4T1 Cells

**Figure 6.**
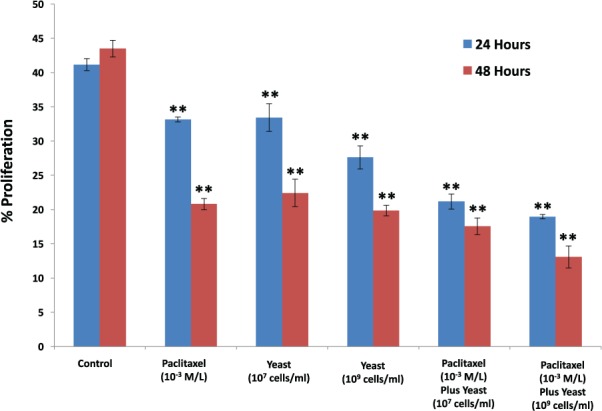
Proliferation of 4T1 cells. The effect of paclitaxel alone (1 × 10^−3^ M/L), and in combination with 2 different concentrations of yeast (1 × 10^7^ and 1 × 10^9^ cells/mL) on the proliferation of 4T1 cells was examined. The percentage proliferation of 4T1 cells was assessed using flow cytometry. Data represent the mean ± SE of 2 experiments performed in triplicate. **P < .01 and was considered as statistically significant.

Figure 6 shows the effect of yeast at two concentrations (1 × 10^7^ and 1 × 10^9^ cells/mL) in the presence and absence of paclitaxel (1 × 10^−3^ M/L) on the percentage of 4T1 cell proliferation. Treatment of 4T1 cells with paclitaxel resulted in inhibition of cell proliferation at 48 hours (50.2*%, P* < .01), as compared with control untreated 4T1 cells. Similarly, 4T1 cell exposure to yeast alone caused a decrease in cell proliferation 48.4%, and 54.4% (*P* < .01) at yeast concentrations 1 × 10^7^ and 1 × 10^9^ cells/mL, respectively, as compared with control untreated 4T1 cells. However, co-culture of 4T1 cells in the presence of yeast plus paclitaxel showed significant inhibition of cell proliferation that was greater than either agent alone.

## Discussion

Paclitaxel is considered to be a common chemotherapeutic drug for the treatment of breast cancer. It induces an apoptotic effect on cancer at high concentrations^33-35^ and, as a result, its treatment is associated with severe side effects, including gastrointestinal, pulmonary, and neuromuscular toxicities, as well as neutropenia, granulocytopenia, and hypotension.^36-38^ Therefore, we have focused our research on finding dietary agents that may have the ability to reduce the toxicity of chemotherapy by using lower drug concentrations while maintaining potency against cancer cells. Our recent studies revealed that arabinoxylan rice bran, Biobran/MGN-3, has the ability to sensitize cancer cells to chemotherapy agents daunorubicin^22^ and paclitaxel in vitro^23^ and in vivo.^21^ In addition, the ability of Biobran/MGN-3 to enhance the effects of interventional therapies for the treatment of hepatocellular carcinoma was examined in a 3-year randomized clinical trial. This previous trial revealed a higher survival rate and a lower percentage of recurrence in patients who received both interventional therapies and Biobran/MGN-3, as compared with chemotherapy alone.^39^ Other dietary products, such as fatty acids, also act as chemosensitizers^40^ by improving the cytotoxic effect of paclitaxel^41^ and increasing the intracellular chemotherapy drug accumulation in cancer cells.^42^ In the current study, we extend the list of chemosensitizing agents to include another dietary agent, baker’s and brewer’s yeast *S. cerevisiae*. Data show that baker’s yeast is able to reduce the concentration of paclitaxel required for killing nonmetastatic and metastatic BCCs in vitro. The IC_50_ value for paclitaxel was significantly reduced against BCCs of human MCF-7, and murine 4T1, and EAC cells in the presence of yeast. Baker’s yeast can therefore potentially be used to reduce the chemotoxic effects of paclitaxel.

Many anticancer drugs function by inducing apoptosis.^1,2^ Paclitaxel induces apoptosis in different cancer cells, including breast cancer,^23^ gastric cancer,^43^ colon cancer,^44^ and leukemia cells^45^ by modifying mitochondrial transition permeability, activating caspase-8 and caspase-3,^44,46^ and Bcl-2 inactivation by a mechanism that may involve the binding of paclitaxel to this antiapoptotic protein.^47^ As demonstrated in this study, heat-killed baker’s yeast also acts as an anticancer agent via induction of apoptosis. These results are in accordance with our earlier studies, which have shown that human breast, tongue, and colon cancer cells undergo apoptosis on phagocytosis of *S. cerevisiae* in vitro.^24-27^ Cancer cells treated with yeast showed clear signs of apoptosis, including nuclear fragmentation and membrane blebbing,^27^ significant decrease in the mitochondrial polarization, and increased activation of caspase-8, -9, and -3 in vitro.^24^ Furthermore, yeast induced extensive apoptosis in nude mice bearing human breast cancer and in mice bearing EAC, as determined by histopathological analysis and by flow cytometry.^28-30^

Earlier studies have also shown that paclitaxel prevents cell proliferation by binding to tubulin in microtubules.^48,49^ This characteristic may explain the observed inhibition of cell proliferation and DNA damage in 4T1 cells treated with paclitaxel. In the present study, we also noted that exposure of 4T1 cells to paclitaxel plus yeast resulted in a marked inhibition of cell proliferation, significant increase in the percentage of DNA damage, and elevation of apoptotic cancer cells. The combined effects of both yeast and paclitaxel were more effective than that of either treatment alone. The underlying mechanisms by which yeast sensitizes cancer cells to chemotherapy are not fully understood, but they might be attributed to yeast’s ability to modulate one or more of the various transport proteins of the ABC superfamily. These proteins are responsible for MDR by decreasing the uptake of the drug or increasing the efflux of the drug from the target organelles. The phytochemical curcumin and phytochemical flavonoids have been described as natural modulators of MDR transporter expression.^19,20^ Curcumin and its metabolite tertrahydrocurcumin were used in restoring drug sensitivity in cancer cells overexpressing the MDR-linked ABC transporters MRP1,^50^ Pgp,^51^ and ABCG2^52^ by directly inhibiting their functions. Similarly, flavonoids have been shown to be potent modulators of major ABC drug transporters.^53,54^

Dietary agents such as Biobran/MGN-3 or the baker’s yeast studied here may provide safe, nontoxic avenues for effective therapies against MDR cancer cells. Studies have shown that yeast is not toxic to nontumorgenic breast epithelial (MCF-10A) cells.^26^ More studies are needed to explore the clinical significance of yeast treatment in different types of cancer. In our ongoing current studies, intratumoral injection of yeast, in combination with low doses of paclitaxel, has been found to significantly reduce tumor size in Ehrlich mammary adenocarcinoma bearing mice, as associated with the development of large degenerative necrotic regions in the tumor (unpublished data). In addition, intratumoral injection of yeast has also been found to be effective in inducing apoptotic effects against skin cancer in rats bearing tumor (unpublished data). We hope that these and other studies will prompt further investments toward studying the effectiveness of yeast treatment for patients with different types of malignancies.

In conclusion, the present study represents the first set of experiments demonstrating that dietary supplementation of baker’s yeast can enhance the apoptotic capacity of paclitaxel in breast cancer cells in vitro. These data suggest that baker’s yeast may be used as an adjuvant for chemotherapy treatment, which may have clinical implications for the treatment of breast cancer.
